# Secondary Carnitine Deficiency in Neonates and Infants Requiring Surgery for Intestinal Obstructions—An Underestimated and Undermanaged Problem

**DOI:** 10.3390/children11020147

**Published:** 2024-01-24

**Authors:** Sheng-Yang Huang, Chia-Man Chou, Hou-Chuan Chen

**Affiliations:** 1 Division of Pediatric Surgery, Department of Surgery, Taichung Veterans General Hospital, Taichung 407219, Taiwan; drugholic@vghtc.gov.tw (S.-Y.H.); donald750410@vghtc.gov.tw (H.-C.C.); 2 School of Medicine, College of Medicine, National Yang Ming Chiao Tung University, Taipei 112304, Taiwan; 3 Department of Post-Baccalaureate Medicine, College of Medicine, National Chung Hsing University, Taichung 402202, Taiwan

**Keywords:** carnitine, ileus, intestinal obstruction, infant, neonate

## Abstract

This study aims to elucidate the relationship between intestinal obstruction and carnitine deficiency in neonates and infants. We retrospectively reviewed medical records of 330 neonates and infants, younger than six months, who underwent surgery for intestinal obstruction at our institute from January 2009 to April 2022. The analysis focused on clinical symptoms, related signs, complications, and etiology of the intestinal obstruction. Tandem mass spectrometry (MS/MS) or urine organic acid analysis was conducted for 47 patients, revealing carnitine deficiency in 16 patients. The incidence of carnitine deficiency was 34.0% in the suspicious group and 4.8% overall, significantly higher than in the general population in Taiwan. Notably, patients with carnitine deficiency experienced prolonged ileus, with a mean fasting duration of 41.7 days (range 7.8–65.5 days), compared to 10.8 days (range 8.2–13.4 days) in patients without carnitine deficiency. Carnitine replacement therapy was administered to twelve patients at dosages ranging from 32 to 90 mg/kg/day. One patient exhibited a drug allergy with skin rashes. Our findings suggest that carnitine deficiency should be considered in cases of neonatal and infant intestinal obstruction. Replacement therapy is straightforward and can be prognostically beneficial for some patients. Therefore, we recommend generalizing MS/MS and urine organic acid analysis, particularly for patients with prolonged ileus.

## 1. Introduction

Carnitine, a quaternary amine compound, plays a crucial role in transporting long-chain fatty acids across mitochondrial membranes for beta-oxidation [1]. The carnitine system encompasses enzymes and transporters governing carnitine synthesis, uptake, and excretion [1]. Carnitine deficiency can manifest as primary, resulting from genetic defects in the carnitine system, or secondary, arising from medical conditions affecting carnitine metabolism or increased carnitine demand [2,3]. Secondary carnitine deficiency is associated with various conditions such as inborn metabolic errors, liver and kidney diseases, and gastrointestinal disorders [4,5]. One condition linked to secondary carnitine deficiency, especially in neonates and infants, is intestinal obstruction, often necessitating surgical intervention [6]. However, the impact of surgery on carnitine metabolism remains poorly understood.

Several studies have reported carnitine deficiency in neonates and infants with intestinal obstruction and surgery [7,8,9]. Nevertheless, the management of carnitine deficiency in this population is frequently overlooked, and the potential effects on clinical outcomes remain underappreciated. This article reviews the literature on carnitine metabolism and deficiency disorders, explores the pathophysiology and clinical characteristics of secondary carnitine deficiency in neonates and infants with intestinal obstruction, and emphasizes the significance of early diagnosis and proper management, including carnitine supplementation, as well as strategies for preventing and monitoring carnitine deficiency in those undergoing surgical interventions.

Carnitine deficiency can be diagnosed through clinical assessment, laboratory tests, and genetic analysis. Clinical symptoms such as muscle weakness, hypoglycemia, and cardiomyopathy can raise suspicion of carnitine deficiency. Laboratory tests, including measurement of serum or plasma carnitine and acylcarnitine levels, offer insights into the body’s fatty acid transport and utilization capabilities. Genetic testing can identify specific mutations associated with carnitine deficiency disorders. Newborn screening programs can detect asymptomatic infants with carnitine deficiency through acylcarnitine measurements in dried blood spots. Confirmatory diagnosis and identification of specific genetic mutations can be accomplished through second-tier molecular tests such as DNA sequencing [5,7]. Distinguishing between primary and secondary carnitine deficiency is a multifaceted process that integrates clinical insights with biochemical and genetic analyses. Biochemically, both conditions manifest as reduced serum carnitine levels, but a more detailed acylcarnitine profile and urine organic acid analysis can offer further clues, such as specific metabolic byproducts indicative of primary metabolic disorders. Genetic testing holds the definitive key, particularly for primary deficiency, by identifying mutations in genes responsible for carnitine transport. Improvement in secondary deficiency is contingent on the treatment of the underlying condition that is causing the depletion or malabsorption of carnitine. Secondary carnitine deficiency may be associated with significantly longer times to commence feeding and longer hospital stays, highlighting the clinical impact of this nutritional deficiency in surgical pediatric populations.

Carnitine replacement therapy serves as the primary treatment for carnitine deficiency patients, with the aim of restoring carnitine levels to normal and enhancing energy metabolism. The specific approach to replacement therapy varies based on the type and severity of the deficiency disorder. In primary carnitine deficiency, resulting from impaired carnitine transport into cells, oral L-carnitine supplementation constitutes the mainstay of treatment. Patients often require lifelong daily supplementation in divided doses to maintain optimal blood levels [3,10]. Secondary carnitine deficiency, stemming from various medical conditions, necessitates addressing the underlying cause alongside carnitine supplementation. This may involve treating the underlying disease, dietary modifications, or adjustments to medications affecting carnitine metabolism. In severe cases, intravenous carnitine supplementation may be required [3,4]. Neonates and infants at risk of carnitine deficiency due to parenteral nutrition may benefit from prophylactic carnitine supplementation to prevent deficiency [9,11]. However, the level of the evidence is not high.

Overall, the relationship between carnitine deficiency and neonatal intestinal obstruction highlights the importance of a comprehensive approach to pediatric care. It underscores the need for routine screening for metabolic disorders in neonates, especially those presenting with intestinal obstruction who are undergoing surgery.

## 2. Materials and Methods

### 2.1. Study Population

A total of 330 neonates and infants under six months of age who underwent surgery for intestinal obstruction at our institute from January 2009 to April 2022 were included. No exclusion was made during the given period for cases fitting the age criteria. All patients underwent routine newborn screening for inherited metabolic disorders according to the national policy. The patients who were suspected to be deficient in carnitine were diagnosed through additional serum amino acid analysis by tandem mass spectrometry (MS/MS) and/or urine organic acid analysis, assessed by pediatric metabolic specialists during formal consultation. Some cases also included serum carnitine level testing. Carnitine replacement was adjusted based on MS/MS results and clinical conditions according to the consultant, either inpatient or outpatient. Medical and surgical histories were reviewed, and clinical outcomes were compared between patients with and without carnitine deficiency. Statistical analysis was performed using MedCalc version 20.110 (Mariakerke, Belgium), employing the chi-square test for categorical variables and the independent t-test for continuous variables. The study was approved by the institutional review board of Taichung Veterans General Hospital without requiring informed consent (consent number: CE22341A).

### 2.2. MS/MS and Urine Organic Acid Analyses

Currently, eleven kinds of congenital metabolic disorders are included in routine newborn screening. Blood samples were collected from infants using a heel prick method and then transferred onto filter paper. The ideal timing for sample collection was set at either 24 h post the infant’s first feeding, or between 48 and 72 h of age. For preterm neonates, defined as those born before 32 weeks of gestation, the protocol was slightly adjusted. While their initial samples were processed in the same manner, a second sample was collected and analyzed one month after birth. Glucose-6-phosphate dehydrogenase deficiency and congenital adrenal hyperplasia were identified most frequently. Other identified conditions include congenital hypothyroidism, phenylketonuria, homocystinuria, galactosemia, maple syrup urine disease, medium-chain acyl-CoA dehydrogenase (MCAD) deficiency, glutaric aciduria type I, isovaleic acidemia, and methylmalonic acidemia. Among them, MCAD deficiency is related to carnitine and fatty acid metabolism; however, its incidence is very low in Asia. The development of MS/MS created the opportunity to diagnose more congenital metabolic diseases earlier. In 2019, primary carnitine deficiency, carnitine palmitoyl transferase (CPT) I deficiency, and CPT II deficiency were included as additional screening items. The blood spots were examined using the PerkinElmer MS/MS spectrometer (model 1445). All sample preparation and mass spectrometry analysis were conducted in strict adherence to the guidelines provided by the equipment manufacturers. Some patients underwent urine organic acid tests for ruling out other possible metabolic disorders. Eliminated residues from intravenous (IV) injection, conservatives, parenteral nutrition, and formulas were often identified. For urine analysis, 5 mL urine was collected by urinary bag. Blood carnitine levels were presented by blood free carnitine (normal range: 4.3–8.5 mg/L) and blood total carnitine (normal range: 6.3–11.6 mg/L), although data collection was not routine. For blood level tests, a 3 mL blood sample was collected, and it was allowed to stand for layer separation. The sample was centrifuged at 2000× *g* for 15 min. After centrifugation, the upper serum layer was carefully aspirated and transferred into a 1.5 mL vial for further analysis.

### 2.3. Carnitine Replacement Therapy

The recommended dose of L-carnitine for selective patients was 32–90 mg/kg/day, and the replacement was mostly administered orally after consultation with experts in pediatric metabolic diseases. The duration of carnitine supplementation depended on fasting periods and feeding conditions and typically did not exceed 6 months. Gradual tapering of the dose was recommended during the replacement period. While IV carnitine is available, it is expensive and not covered by National Health Insurance for secondary carnitine deficiency. For patients with malabsorption from the gastrointestinal tract or short bowel syndrome, the IV form provides the possibility for replacement. However, due to the issue of cost and a lack of previous experience, it is less commonly used than oral supplementation in the institute.

## 3. Results

### 3.1. Patient Characteristics

Patients with prolonged ileus and unscheduled fasting were suspected to have carnitine deficiency or other metabolic disorders. MS/MS or urine organic acid analysis was performed for forty-seven such patients (14.2%), and carnitine deficiency was found in sixteen patients. The positive rate of MS/MS was 34.0%, and the overall incidence was 4.8%. The high positive rate of 34.0% (16/47) revealed that more patients might be concealed in the group with intestinal obstruction or ileus. The demographic data and characteristics of the study population are summarized in Table 1. Only one patient was diagnosed with primary carnitine deficiency. No differences in gender, age at surgery, comorbidity, inborn error screening tests, and serum ammonium level were found between patients with or without carnitine deficiency. The most common comorbidity was prematurity and small for gestational age in both groups, which was 37.5% for patients with carnitine deficiency and 31.8% for patients without carnitine deficiency.

### 3.2. Perioperational Parameters and Outcomes

As shown in Table 2, the symptom duration of intestinal obstruction before and after surgery were not different between these two groups. The post-operative fasting period and hospital stay were considerably longer in patients with carnitine deficiency. This can be blamed on prolonged periods of inability to feed causing a lack of carnitine, namely secondary deficiency [6]. The surgical complication rate was higher in the carnitine deficiency group, however, without statistical difference. In that, the most common surgical complications were wound infection (1.2%), massive intra-abdominal bleeding (1.2%), adhesion ileus (2.4%), and anastomotic stricture (1.5%). Although no difference in long-term complication rate was observed in both groups, developmental delay was the most frequent sequela (14.8%) in all patients. No mortality was encountered in patients with carnitine deficiency. Those without carnitine deficiency died of the underlying disease, and the most common cause of death was prematurity, congenital heart disease, and sepsis, in that order. Mean follow-up duration was similar in both groups.

### 3.3. Surgical Diagnoses of Suspected Metabolic Disorder and Carnitine Deficiency

**Figure 1:**
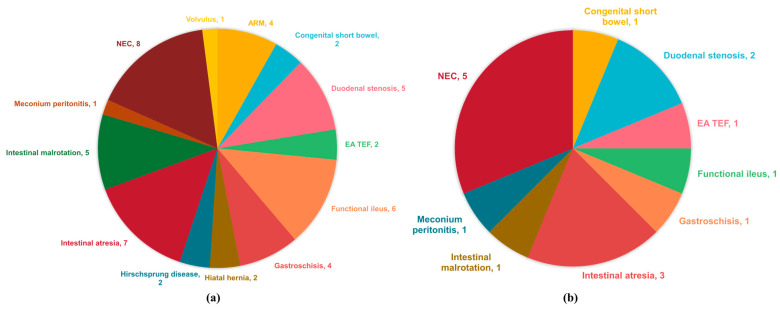
The diagnoses of the case series. (**a**) The diagnoses of patients with suspected metabolism disorders underwent additional MS/MS or urine organic acid analysis. Note that one patient had simultaneously intestinal malrotation, hiatal hernia, and duodenal stenosis. (**b**) The diagnoses of patients with confirmed carnitine deficiency. ARM, anorectum malformation. EA TEF, esophageal atresia with tracheoesophageal fistula. NEC, necrotizing enterocolitis.

In our study, we closely examined the incidence of suspected metabolic disorders in infants undergoing pediatric surgery, focusing particularly on those who underwent MS/MS and urine organic acid analysis. Figure 1a illustrates the distribution of diagnoses among the 47 patients evaluated. The spectrum of surgical diseases encountered was diverse, with necrotizing enterocolitis (NEC), intestinal atresia, functional ileus, intestinal malrotation, and duodenal stenosis being the most prevalent. Notably, one patient with Klinefelter syndrome presented a unique case with multiple surgical issues, including intestinal malrotation, hiatal hernia, and duodenal stenosis, which underscores the complexity of metabolic disorders in the context of congenital anomalies.

The data on patients with confirmed carnitine deficiency, depicted in Figure 1b, further highlights the correlation between certain surgical conditions and metabolic disorders. Of these, five patients diagnosed with NEC, three with intestinal atresia, and two with duodenal stenosis were found to have carnitine deficiency. This pattern suggests a potential link between specific types of surgical intestinal disorders and the risk of developing carnitine deficiency. Moreover, a rare case of primary carnitine deficiency was observed in a patient with meconium peritonitis secondary to jejunal atresia with intrauterine perforation, indicating the varied and complex nature of these conditions.

### 3.4. Responses to Carnitine Replacement

In our cohort of patients with confirmed carnitine deficiency, a significant portion, twelve out of sixteen (75.0%), received prescribed carnitine replacement therapy. This intervention was notably more common in patients with prolonged fasting periods, averaging 50 days, compared to those who did not receive supplementation, who had an average fasting period of 13.8 days. The detailed information regarding the administration of carnitine, including dosage and duration, is meticulously documented in Table 3 of our study. The levels of blood free and total carnitine in these patients were notably low, with average levels of 1.24 mg/L and 3.15 mg/L, respectively, both of which fall below the normal range of 4.3–8.5 mg/L and 6.3–11.6 mg/L. The prescribed dosage of carnitine replacement varied, ranging from 32 to 90 mg/kg/day, tailored to individual patient needs and conditions. This wide range in dosage reflects the personalized approach taken in administering the supplement, considering factors such as the severity of deficiency and patient tolerance.

Interestingly, one patient in our study exhibited an allergic reaction to oral carnitine, manifesting as skin rashes. This rare occurrence highlights the need for careful monitoring of patients during carnitine supplementation, especially considering potential adverse reactions. Despite this, all patients demonstrated normalized MS/MS results in the follow-up period after receiving carnitine replacement, indicating the effectiveness of the treatment in correcting the deficiency. Our study did not observe any complications or sequelae related to carnitine deficiency in the patient cohort. This outcome suggests that with appropriate and timely treatment, the adverse effects of carnitine deficiency can be effectively mitigated.

**Figure 2:**
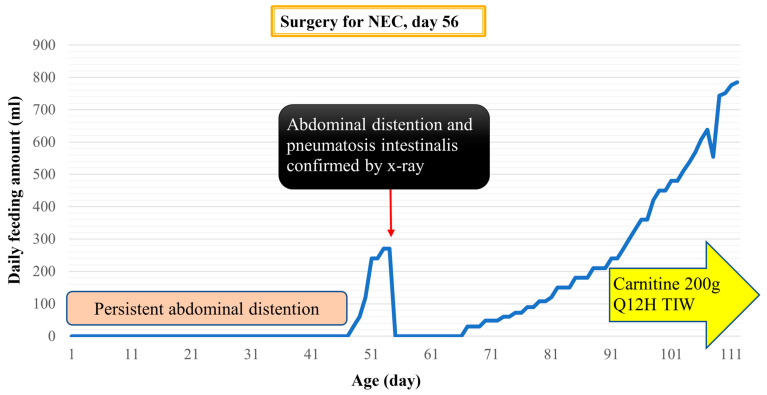
The feeding condition of one patient with carnitine deficiency. The response to oral carnitine replacement of a patient with NEC and confirmed carnitine deficiency. After adding carnitine on the 91st day of life (36 days after surgery), the feeding amount could be increased faster.

The response to carnitine replacement was particularly noteworthy in one patient with NEC, as depicted in Figure 2. This patient’s case exemplified the potential impact of carnitine supplementation on clinical outcomes. The patient experienced persistent ileus for 36 days post-operation, but notably, enteral feeding was successfully resumed shortly after the initiation of carnitine replacement. The administration of carnitine via orogastric tube was initialized with small amounts of medication dissolved in distilled water (1 or 2 mL every time). This observation underscores the potential therapeutic benefit of carnitine in patients with prolonged ileus and suggests a possible role for carnitine supplementation in accelerating recovery in such cases.

## 4. Discussion

The overall incidence of surgical intestinal obstruction in this series was 4.8%. In addition, the incidence of primary carnitine deficiency was 0.3% (1/314), which was higher than in the reported data. The incidence of carnitine uptake deficiency in newborns was 1 in 67,000 by newborn screening reports in Taiwan [5]. CPT I, CPT II, and carnitine-acylcarnitine translocase (CACT) deficiencies jointly affected 0.38 in 100,000 in another local study [6]. A Taiwanese series by second-tier molecular tests revealed an incidence of 1 in 30,237 [7], which was only 1.1% of our data in patients with surgical intestinal obstruction. In a Qatar report, 8.56% of extremely preterm neonates (864 patients) have secondary carnitine deficiency due to malnutrition [8]. In a US national study among members of the American Academy of Pediatrics Section on Perinatal Pediatrics, low clinical awareness was the major reason for low identification [9]. The same observation could explain the higher incidence in the current cohort for the routine additional MS/MS for patients with prolonged fasting or poor recovery from surgery.

According to the findings of this study, secondary carnitine deficiency should be a concern for neonates and infants with intestinal obstruction, especially for those with upper gastrointestinal obstruction, necrotizing enterocolitis, congenital short bowel syndrome, and prolonged fasting periods. It was found that all patients with confirmed carnitine deficiency had upper gastrointestinal tract obstruction but not colorectal obstruction. Among patients with suspected carnitine deficiency, higher positive rates were observed in conditions such as NEC (5 out of 8 patients), congenital short bowel syndrome (1 out of 1 patient), duodenal stenosis (2 out of 5 patients), esophageal atresia with tracheoesophageal fistula (1 out of 2 patients), intestinal atresia (3 out of 7 patients), and meconium peritonitis (1 out of 1 patient). Conversely, lower rates were noted in conditions such as functional ileus (1 out of 6 patients), gastroschisis (1 out of 4 patients), intestinal malrotation (1 out of 5 patients), anorectal malformation (0 out of 4 patients), and Hirschsprung disease (0 out of 2 patients). The faster resumption of feeding in patients with anorectal malformation and Hirschsprung disease may account for the prevention of carnitine deficiency in these conditions. Though oral replacement therapy after resuming intake is simple and has a favorable prognosis, IV form of carnitine replacement may help patients without any chance of gastrointestinal intake. Currently, there is no solid evidence for the use of IV L-carnitine supplements for total parenteral nutrition for prophylaxis.

Carnitine deficiency could result in multiple-organ manifestations in the first year of life after prolonged fasting or severe infections. Progressive cardiomyopathy, hypoketotic hypoglycemic encephalopathy, and skeletal myopathy are common related diseases. These three major conditions did not occur in this series. Other conditions, such as hepatomegaly, elevated liver enzyme, hyperammonemia, gastrointestinal dysmotility, hypochromic anemia, recurrent infections, and developmental delay, might also occur as complications [3]. Three patients with carnitine deficiency had developmental delays of different degrees in the study period. Two of the three patients were preterm. All three patients had normal repeated MS/MS 3–4 months after carnitine replacement. The development delay might be multifactorial. Oral L-carnitine at 100–200 mg/kg/day is highly effective (15% bioavailability), and early identification with timely treatment may prevent adult-onset manifestations [10]. The lower dosage in the current study (32–90 mg/kg/day) for most patients was because most patients had secondary deficiency due to prolonged fasting. The possible obscure side effects, such as stomach upset, headache, muscle pain, muscle weakness, and fishy body odor, may further compromise the compliance of parents. The administration frequency (once, twice, or three times daily) seems less important than the total daily dosage. The IV form of carnitine may have less side effects but higher costs. Currently, IV carnitine has not been frequently used in neonates or infants. In a systemic review by Olid et al., IV supplementation in parenteral nutrition (PN) increases the carnitine levels but has no effect on lipid profile, weight gain, morbidity, mortality, or hospital stay [11].

Our study identified a notable prevalence of carnitine deficiency in patients with prolonged ileus and unscheduled fasting, highlighting a possible underestimation in the group with intestinal obstruction or ileus. While we reported a 34.0% positive rate of carnitine deficiency (16/47), and an incidence of 0.3% (1/314) in surgical intestinal obstruction cases, these findings should be interpreted with caution due to certain limitations. First, our study’s small sample size limits the generalizability of these findings. Larger, multi-center studies are needed to confirm these rates and to explore the causal relationship between intestinal obstruction and carnitine deficiency more robustly. Second, the higher incidence of primary carnitine deficiency in our cohort compared to previous Taiwanese reports [6,7] may be influenced by our routine additional MS/MS testing for patients with prolonged fasting or poor recovery from surgery. The current study did not include patients without intestinal obstruction requiring surgery for comparison, therefore, this observation requires further validation to support its significance.

We observed a range of conditions associated with higher rates of suspected carnitine deficiency, such as NEC, congenital short bowel syndrome, and upper gastrointestinal tract obstruction. However, these associations should not be seen as definitive due to the absence of a control group and the potential for confounding factors in our retrospective study design. Moreover, the benefits of carnitine supplementation, while suggested, were not conclusively demonstrated due to the lack of a control group and reliance on observational data. The potential benefits of carnitine supplementation, particularly oral L-carnitine, are promising but require more rigorous investigation. Our findings indicate a favorable prognosis with oral replacement therapy with low minor complication rate. Our study also touches upon the multifactorial nature of developmental delays in patients with carnitine deficiency. While early identification and treatment may prevent more severe manifestations, our limited dosage range and lack of a control group make it difficult to draw firm conclusions about the efficacy of the treatment.

The study on carnitine deficiency in neonates and infants with intestinal obstruction is pivotal in bridging several crucial gaps in medical research and clinical practice. It sheds light on the intricate link between carnitine deficiency and neonatal intestinal obstruction, an area previously not explored in depth. This research provides critical insights into how these two conditions interrelate and the impact of surgical interventions on carnitine metabolism in this vulnerable group. The research also accentuates the importance of preventive measures and monitoring strategies, especially for infants at risk due to parenteral nutrition or metabolic stress. Overall, this study is a vital step forward in guiding future research, informing clinical practices, and improving healthcare outcomes for neonates and infants with these complex medical needs. The research on carnitine deficiency in neonates and infants with intestinal obstruction is a multifaceted field that offers numerous prospects for future studies and practical applications. It paves the way for longitudinal studies aimed at understanding the long-term health outcomes in this demographic, providing valuable insights into treatment effectiveness and ongoing health challenges. At the genetic and molecular level, this research could unravel the intricate mechanisms behind carnitine deficiency, potentially leading to more targeted therapies through the identification of specific genetic mutations [12,13]. Additionally, expanded screening programs including carnitine level tests could lead to earlier detection and intervention in at-risk infants. The development and evaluation of specialized nutritional interventions and formulas are also crucial, especially for those unable to feed orally due to intestinal obstructions.

Moreover, this area of research opens doors to pharmacological advancements, including the development of more effective supplementation forms, and explores the differential impact of carnitine deficiency across various infant populations. Preventive strategies in hospital settings and the implementation of monitoring and supplementation protocols for at-risk populations are vital aspects of this research. The findings could significantly influence healthcare policies and the development of clinical practice guidelines, improving the management of carnitine deficiency. Additionally, educational programs for healthcare providers and parents could raise awareness about the signs, consequences, and management of this condition. Lastly, the development of new diagnostic tools and technologies could revolutionize the quick and accurate diagnosis of carnitine deficiency in clinical settings, potentially leading to improved outcomes for affected infants.

## 5. Conclusions

In conclusion, this study highlights the critical need for attention to secondary carnitine deficiency in neonates and infants with intestinal obstruction, particularly those with conditions like upper gastrointestinal obstruction, necrotizing enterocolitis, congenital short bowel syndrome, and prolonged fasting periods. The administration of oral carnitine supplements post-resumption of intake presents a straightforward and effective approach with a positive prognosis. However, the efficacy of intravenous prophylactic L-carnitine supplements in parenteral nutrition remains unsubstantiated by solid evidence. The current study supports the use of IV carnitine only in scenarios where oral administration is not feasible or tolerated. Our findings advocate for a comprehensive approach to managing pediatric patients with intestinal obstructions, emphasizing the importance of early screening for metabolic disorders like carnitine deficiency. Additionally, these insights call for continued research to better understand the optimal methods of carnitine supplementation, whether oral or intravenous, and to establish evidence-based protocols for treatment. The broader goal remains to improve the outcomes and quality of life for these vulnerable pediatric patients through timely and effective intervention strategies.

## Figures and Tables

**Table 1:** Characteristics of the study population.

	With Carnitine Deficiency	Without Carnitine Deficiency	*p*
Patient number	16	314	
Female	8 (50.0%)	115 (36.6%)	0.281
Age * (day)	23.8 (5.1–42.4)	30.6 (26.5–34.7)	0.468
Comorbidity	7 (43.8%)	151 (48.1%)	0.735
Abnormal inborn error ** screening	1 (6.3%)	53 (16.9%)	0.263
CAH	1 (6.3%)	36 (11.5%)	0.519
G6PD deficiency	0	11 (3.5%)	0.447
Homocystinuria	0	2 (0.6%)	0.749
Isovaleric acidemia	0	3 (1.0%)	0.694
Glutaric aciduria-1	0	1 (0.3%)	0.821
Hypothyroidism	0	3 (1.0%)	0.694
Methylmalonic acidemia	0	1 (0.3%)	0.821
MSUD	0	1 (0.3%)	0.821
Galactosemia	0	1 (0.3%)	0.821
Serum ammonia (ug/dl) *	123.1 (57.1–189.1)	121.6 (95.2–148.0)	0.958

CAH: congenital adrenal hyperplasia; G6PD: Glucose-6-phosphate dehydrogenase; MSUD: maple syrup urine disease. * Mean (95% confidence interval). ** One patient may have multiple positive results.

**Table 2:** Peri-operational parameters and outcomes.

	With Carnitine Deficiency	Without Carnitine Deficiency	*p*
Patient number	16	314	
Symptom duration * (day)	13.4 (2.4–24.5)	18.0 (14.5–21.5)	0.567
Time to feed * (day)	41.7 (17.8–65.5)	10.8 (8.2–13.4)	<0.0001
Hospital stays * (day)	76.3 (44.6–107.9)	33.4 (28.2–38.5)	0.0004
Surgical complication **	3 (18.8%)	23 (7.3%)	0.097
Long-term complication **	3 (18.8%)	56 (17.8%)	0.925
Mortality	0	21 (6.7%)	0.221
Mean follow (months) *	44.1 (29.7–58.5)	52.3 (48.3–56.3)	0.369

* Mean (95% confidence interval). ** One patient may have multiple positive results.

**Table 3:** Details of carnitine replacement.

Item	Data
Patients with replacement	12 (75.0%)
Serum free carnitine level * (mg/L)	1.24 (0.51–1.97)
Serum total carnitine level * (mg/L)	3.15 (1.55–4.74)
Dose and frequency	50 mg/kg, daily, or 16–45 mg/kg, twice daily, or 20–30 mg/kg, three times daily
Duration * (month)	3.8 (2.8–4.9)
Drug allergy	1 (6.3%)
Response (followed MS/MS)	All normal
Carnitine deficiency-related complications	None

* Mean (95% confidence interval).

## Data Availability

The data presented in this study are available on request from the corresponding author. The data are not publicly available due to the policy of institution’s IRB.
